# Postoperative Delayed Facial Nerve Palsy: A Surgeon's Nightmare

**DOI:** 10.7759/cureus.58691

**Published:** 2024-04-21

**Authors:** Gulistan Bano, Sumeet Angral, KSBS Krishna Sasanka, Swaha Panda, Ajit Kumar, Saurabh Varshney, Sree Sudha Tanguturi Yella, Pradosh Kumar Sarangi

**Affiliations:** 1 Otolaryngology-Head and Neck Surgery, All India Institute of Medical Sciences, Deoghar, Deoghar, IND; 2 Otolaryngology-Head and Neck Surgery, All India Institute of Medical Sciences, Bilaspur, Bilaspur, IND; 3 Pharmacology, All India Institute of Medical Sciences, Deoghar, Deoghar, IND; 4 Radiodiagnosis, All India Institute of Medical Sciences, Deoghar, Deoghar, IND

**Keywords:** csom: chronic suppurative otitis media, steroid treatment, postop complication, delayed facial nerve palsy, cortical mastoidectomy

## Abstract

The facial nerve supplies motor, sensory, and parasympathetic innervation to the head and neck, and its paralysis can have significant physical and psychological impacts. This study discusses a compelling case involving a 21-year-old male who developed delayed facial nerve palsy (DFNP) on the eighth day after cortical mastoid surgery. Through conservative management, the patient achieved a full recovery by the 52nd day. Our experience underscores the importance of approaching DFNP with patience, emphasizing the need for thorough counseling of both the patient and their family members.

## Introduction

The ability to convey emotions through facial expression is a highly cherished attribute for humans. Facial muscle paralysis can lead to significant disfigurement, resulting in profound psychological and emotional distress for those affected. The facial nerve plays a crucial role in providing motor, sensory, and parasympathetic innervation to the head and neck [1]. The potential physical and psychological impact of facial nerve paralysis on functionality and appearance can be devastating. The treating physician should prioritize a comprehensive history and thorough examination to effectively address the condition. Facial palsy is a common immediate postoperative complication of ear surgeries. However, delayed facial nerve palsy (DFNP) is an infrequent occurrence associated with ear surgeries. It manifests after more than 72 hours following an uneventful ear surgery. Shea coined the term "five and a half day syndrome," noting that all instances of DFNP in his personal observations exhibited a consistent time lag from the surgery [1].

## Case presentation

A 21-year-old patient underwent right cortical mastoidectomy with tympanoplasty in our hospital for right chronic suppurative otitis media (CSOM), mucosal type with moderate hearing loss, with sclerosed mastoid without any complications. The patient's hospital stay was uneventful, with no facial palsy during the postoperative period until the fourth day of hospitalization. The patient was discharged in stable conditions. Follow-up was done on the eighth postoperative period, and sutures were removed. Until this time, the patient was in stable condition with intact facial nerve functions. However, in the evening of the eighth postoperative period, the patient suddenly developed right-sided facial weakness with deviation of the angle of mouth, incomplete closure of the right eye, and loss of forehead wrinkling. On his visit to the hospital on the ninth postoperative day, he was diagnosed with Grade IV House Brackmann (HB) facial nerve palsy. The patient had no history of fever during the postoperative period nor any history suggestive of varicella-zoster infection in childhood.

On otoscopic examination, AbGel was seen in the external auditory canal, and the postauricular wound was healthy. The rest of the ENT examination was normal. The patient was readmitted to the hospital, and routine blood investigations (CBC, liver function test (LFT), renal function test (RFT), and blood sugar) were done, which were unremarkable. Varicella-zoster antibody (anti-VZV) titer could not be done because of the unavailability of this test in the hospital and peripheral areas. The patient was started on antivirals (Tab valacyclovir 500 mg three times a day (TDS) for five days, broad-spectrum antibiotics, steroids in tapering dose (oral Tab prednisolone) and injectable (Inj Dexa), anti-inflammatory, proton pump inhibitors (PPIs), Tab methylcobalamin (1500 mg), along with eye care measures and facial physiotherapy.

**Figure 1:**
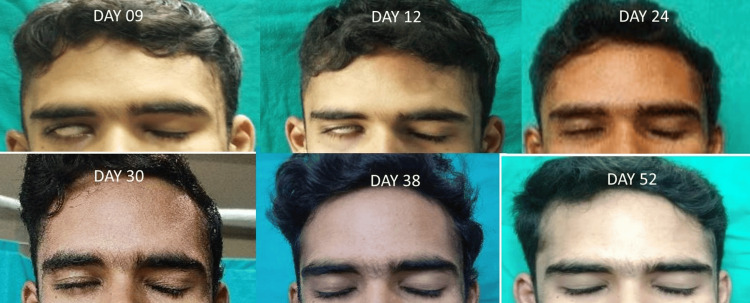
A series of pictures depicting events of eye closure in a patient with DFNP. DFNP: delayed facial nerve palsy.

The patient's mother displayed emotional distress, as her son is the sole male offspring in their family. The manifestation of facial palsy was unprecedented within their familial history. Regular counseling was done to both the patient and the attendants, and the nature of the disease was explained to the patient. The patient started recovering from right facial nerve palsy on postoperative day 17 with initial improvement in eye closure, which was completed by day 30 (Figure 1).

**Figure 2:**
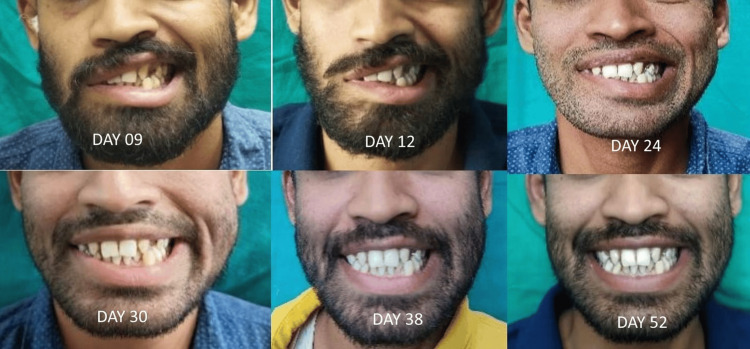
A series of pictures depicting the progression of changes in the angle of the mouth in a patient with DFNP. DFNP: delayed facial nerve palsy.

The improvement in the deviation of angle of mouth started on day 24 and was completed on day 52 (Figure 2).

**Figure 3:**
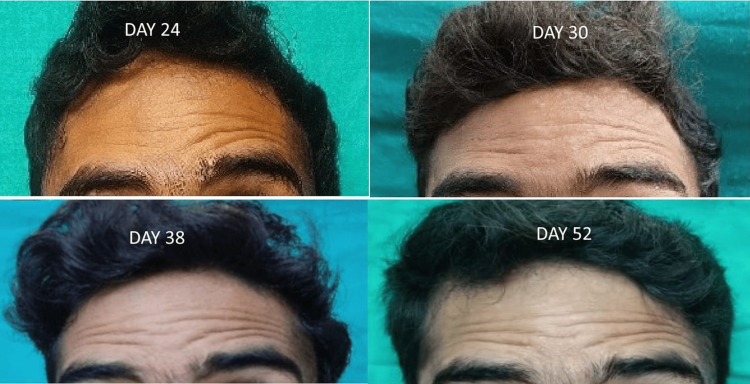
A series of pictures depicting events of forehead furrowing in a patient with DFNP. DFNP: delayed facial nerve palsy.

The gradual appearance of forehead furrowing over the right side started on day 17, which was completed on day 38 (Figure 3).

The complete facial recovery took 52 days. Neotympanum was also completely healed by day 52. The case timeline is presented in Table 1.

**Table 1:** Case timeline and events of the patient. HB grade: House Brackmann grade; PPI: proton pump inhibitor; EAC: external auditory canal; CMC: carboxymethyl cellulose; RBS: random blood sugar; BD: twice a day.

Postoperative day	Facial nerve function	Treatment prescribed	Remarks	HB grade
Day 1	Intact	IV antibiotics, PPI, anti-inflammatory, antihistaminic	Facial nerve intact	-
Day 3	Intact	Oral antibiotics, anti-inflammatory, antihistaminic	Patient was discharged in stable conditions	-
Day 8 (morning)	Intact	Ear drops started. Dry ear precautions. Antihistaminic	Suture removal was done. AbGel was present in EAC	-
Day 8 (evening)	Right-sided facial weakness		Patient planned to visit the hospital	-
Day 9	Diagnosed with grade IV right facial nerve palsy	Tab prednisolone Tab valacyclovir Tab ciprofloxacin Tab methylcobalamin Tab pantoprazole Tab Chymoral Forte Tab montelukast + levocetirizine Ciplox D ear drop CMC eye drop Facial exercises Eye patching	Patient admitted to the hospital. Counseling done	IV
Day 12	Status quo	Status quo	Status quo	IV
Day 14	Status quo	Inj Dexamethasone 2 ml BD started for three days, then 1 ml BD for the next three days, and then 0.5 ml BD for the next six days	RBS monitoring	IV
Day 17	Improvement started with raising eyebrows and closing the eye with mild effort	Dexa 1 ml	RBS monitoring	IV
Day 24	Complete closure of the eye with minimal effort, mild improvement in raising eyebrows, and improvement in the deviation of the angle of mouth	Inj. Dexa 0.5 ml Tab prednisolone, Tab pantoprazole Tab methylcobalamin Facial exercises, eye patching, eye and ear drop	Patient discharged	III
Day 30	Complete closure of the eye with minimal effort, moderate improvement in raising eyebrows, and improvement in the deviation of the angle of mouth	Tab prednisolone, Tab methylcobalamin Facial exercises, Eye drop		III
Day 38	Only mild deviation of the angle of mouth		Facial exercises Methylcobalamin	II
Day 52	Full recovery of facial nerve function		Dry ear precautions, neotympanum intact	I

## Discussion

Facial nerve palsy may manifest either immediately or with a delay after surgery. Immediate palsy could be attributed to the use of local anesthetics for infiltrations, and it often resolves within a few hours. On the other hand, delayed facial palsy presents its symptoms several days or even weeks after the surgical procedure.

DFNP exhibits a bimodal onset with two peaks, characterized by early and late occurrences [2]. The early onset of DFNP typically occurs within three to five days, likely attributed to neural edema, particularly in the meatal foramen. It has been proposed that the generation of heat and/or inflammation caused by the drilling of the temporal bone during the mastoidectomy procedure may indirectly lead to intratubal facial nerve edema. In contrast [3], the exposure of the facial nerve and/or chorda tympani nerves within the operative field could trigger the reactivation of the herpes virus, potentially explaining the relatively delayed onset of postoperative symptoms. Shea noticed in a large series of stapedectomies that DFNP occurred from five to 16 days (mean 8) after surgery [4]. In our case, the onset of facial palsy was on the eighth postoperative day. One of the long-delayed onsets is the case report by Gyo and Honda, when the palsy was noticed on the 14th postoperative day [5].

Currently, the most widely accepted theory explaining delayed facial palsy involves the reactivation of dormant viruses [6]. Edema, leading to the compression of nerve fibers, is typically the cause of reversible delayed facial paralysis [7] or decreased blood supply to facial nerve, which may be due to heat or inflammation during the drilling process, causing palsy in the early postoperative period [8]. The thermal impact of the drill and the lifting of the tympanomeatal flap have the potential to reactivate dormant viruses residing within the ganglion geniculi, facial nerve, or facial nuclei. This phenomenon is frequently observed following acoustic neuroma surgeries (2.2-29%). Additionally, it has been documented after vestibular neurectomy (0-18%), stapes surgery (0.5-1%), endolymphatic sac surgery (1%), cochlear implantation surgery (0.4-0.7%), and mastoidectomy (0.38-1.4%) [9]. Vrabec reported two patients with DFNP in conjunction with drainage from the postauricular wound, and he assumed that bacterial infection with inflammation around the nerve was the suspected mechanism for facial dysfunction [2].

The reactivation of viruses can occur during episodes of general temporary suppression of the immune system induced by physical or emotional stress, simultaneous bacterial or viral infections, neoplasms, and mechanical or surgical trauma, including localized surgical stress [1]. In cases of DFNP, the literature has reported a specific rise in serum anti-VZV IgG antibodies, validating the suspicion of viral reactivation [10,4]. However, we could not conduct this test due to its unavailability at our center at the present moment as well as due to financial constraint of the patient. Surgical trauma, along with the induced immunosuppression post-surgery and the administration of exogenous steroids, can create a predisposition to the reactivation of viruses that typically remain dormant in the geniculate ganglion [11].

Steroids play a crucial role as the primary approach in managing this condition. Some studies have reported partial success with intraoperative decompression of the meatal foramen. Acyclovir has been recommended by some, especially as prophylaxis for delayed facial palsy in patients with a history of viral activation [12]. Safdar et al. recommended a standard postoperative steroid regimen of 4 mg of dexamethasone administered every six hours, with a gradual tapering over a period of five to seven days following the surgery [13].The effectiveness of steroids and antivirals in treating idiopathic facial nerve palsy is well established and supported by numerous studies. The good results were achieved when patients were started on prednisone and acyclovir within three days of onset [14]. In our patient, based on the complete recovery of facial nerve function, the combined use of acyclovir and prednisone proved to be an effective treatment in the management of DFNP.

Alternatively, there is a strong likelihood that prophylactic antiviral treatment could effectively prevent the majority of cases of DFNP. The facial canal decompression, mainly the labyrinthine segment, has also been proposed as a technique for preventing DFNP in vestibular surgeries. The duration of recovery following the onset of DFNP varies between two and 270 days, with a mean of 45±43 days. All reported cases achieved a final HB grade of I-II [14], irrespective of the treatment modalities employed.

The prognosis is typically favorable when facial palsy does not advance to full paralysis. In cases of complete paralysis, the prognosis varies widely, ranging from eventual normal function to permanent total paralysis. The variability in prognosis for complete paralysis may be attributed to edema around the facial nerve resulting from surgery. Steroids constitute a crucial first-line approach in managing this condition.

However, there is no clear-cut guideline for managing this emergency. Timely and supportive presentation to the otolaryngologist, along with appropriate supportive treatment for the right duration of time, is of paramount importance in alleviating symptoms and providing relief to the patient. Methylcobalamin was also administered to our patient. Additionally, the patient engaged in daily facial exercises, performing them five to six times a day for 20 min each, for one month. Overall, the prognosis for individuals experiencing DFNP is highly favorable, with approximately 88% expected to recover to their initial grade or achieve even better outcomes [15]. Patients with DFNP tend to exhibit better recovery of facial nerve function compared to those with immediate onset paralysis, and incomplete paralysis is associated with a higher likelihood of achieving a normal recovery.

## Conclusions

DFNP is a rare occurrence that can stem from various factors, including surgical stress-induced virus reactivation, intraoperative trauma, or chorda tympani nerve laceration, leading to retrograde facial nerve edema. While these factors may provoke the condition, it generally carries a favorable prognosis and recovery rate when managed conservatively by an otolaryngologist. Collaboration between otolaryngologists and physiotherapists plays a crucial role in facilitating timely patient improvement. Treatment protocols, as outlined in the literature, primarily emphasize the early initiation of systemic steroids and antivirals. In most cases, patients exhibit a positive prognosis, with facial nerve function typically returning to near-normal levels. Based on our experience, managing DFNP requires patience and comprehensive counseling for both the patient and their family members. Nonetheless, larger-scale studies across multiple centers are needed to establish detailed encounters and formulate guideline algorithms for effective management.

Declaration of patient consent

The authors certify that they have obtained all appropriate patient consent forms. In the form, the patient has given his consent for his images and other clinical information to be published in a scientific journal for sharing experience and knowledge and for improving the patient’s care. The patient understands that his name and initials will not be published, and due efforts will be made to conceal his identity, but anonymity cannot be guaranteed.
